# Epilepsia Partialis continua secondary to diabetic ketoacidosis

**DOI:** 10.1002/ccr3.6701

**Published:** 2022-12-05

**Authors:** Ahmed Mahmoud Sayed Sayedahmed, Abdelrahman Abdelgader Mohammed Alkhair Ebrahim, Abdalla Mohamed Abdalla Mohamedali, Mahmoud Saeed Saad Mahgoub

**Affiliations:** ^1^ Haj El‐Safi Teaching Hospital Omdurman Islamic University Omdurman Sudan; ^2^ Trauma & Orthopedics Department Omdurman Islamic University Omdurman Sudan; ^3^ Haj El‐Safi Teaching Hospital University of Khartoum Khartoum Sudan

**Keywords:** diabetes, diabetic ketoacidosis, epilepsia partialis continua, metabolic, seizures

## Abstract

Epilepsia partialis continua is a rare epileptic syndrome presenting with simple, partial, repetitive motor seizures. It can be a result of structural central nervous system lesion or due to metabolic causes. We report a case of a 65‐year‐old male patient diagnosed with EPC secondary to diabetic ketoacidosis.

## Graphical abstract

Although epilepsia partialis continua is a rare presentation and its occurrence secondary to diabetic ketoacidosis is even a rarer presentation, the diagnosis should be considered in patients with diabetic ketoacidosis who have no other metabolic abnormalities and a normal central nervous scan.

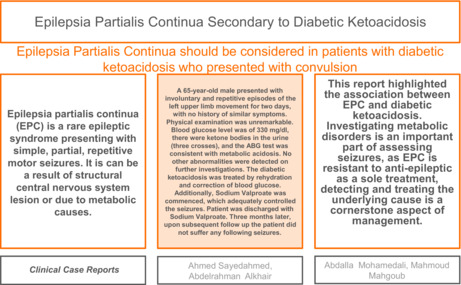

## 1 INTRODUCTION

Epilepsia partialis continua (EPC) is a disease characterized by simple partial motor seizures that are limited to a one part of the body in the form of repetitive regular or irregular clonic jerks without loss of consciousness.^1^ EPC is a rare disease, with an estimated prevalence of one per million in the United Kingdom.^2^ Additionally, EPC is reported to be more common among males.^3^

EPC can occur as a result of structural damage, neoplasia, cortical dysplasia, metabolic abnormalities, or infection.^4^ Seizures can continue for days, weeks, or potentially years, remaining localized to a single part of the body.^1^ EPC management is in the form of antiepileptics as well as identification and treatment of the underlying cause.^5^

Diabetes mellitus (DM) can cause central nervous system (CNS) complications, in form of cognitive dysfunction, metabolic and vascular complications. Regarding cognitive impairment, DM was reported to be associated with Alzheimer's disease.^6^, ^7^ In addition, patients with DM have a higher risk of stroke (2.5–3.5 times) when compared to non‐diabetic individuals.^8^ In terms of metabolic consequences, hypoglycemia causes a range of symptoms—ranging from cognitive impairment to coma and brain damage.^9^ Finally, non‐ketotic hyperglycemic status (NKH) and diabetic ketoacidosis (DKA) were reported to be causes of EPC.^1^, ^10^ However, the occurrence of EPC in patients with DKA is a rare phenomenon.^10^, ^11^, ^12^ We present a case report of EPC secondary to diabetic ketoacidosis.

## 2 CASE PRESENTATION

A 65‐year‐old male patient presented to the emergency department due to involuntary and repetitive episodes of the left upper limb movement for 2 days. These episodes consisted of continuous clonic movement of the left elbow, left wrist, and fingers of the left hand. Seizures had been occurring every 2–5 h, continuing for one to 3 min in each episode. To add, consciousness and sphincter control were preserved throughout the episodes. The patient denied a history of similar symptoms or epilepsy.

The patient looked unwell, temperature was 37.2°C, pulse rate was 98 beats per minute, respiratory rate was 28 cycles per minute, and blood pressure was 100/70 mmHg. In addition, the patient was thirsty with dry lips and mouth. The neurological examination of the patient was normal.

Based on the presentation, central nervous system lesions and electrolyte abnormalities were considered as a differential diagnosis. Based on that, laboratory investigations, magnetic resonance imaging (MRI), cerebrospinal fluid (CSF) study, and an electroencephalogram (EEG) were ordered.

Laboratory investigations revealed a glucose level of 330 mg/dL (normal value: 70–100 mg/dL) and ketone bodies in the urine (three crosses). An arterial blood gas (ABG) test was consistent with metabolic acidosis (Ph: 7.24, pCO_2_: 22.1 mmHg, HCO_3_: 16 mEq\L) (normal values; PH: 7.35–7.45; PCO_2_: 35–45 mmHg; HCO_3_: 22–26 mEq/L). This established a new diagnosis of DM and diabetic ketoacidosis. Furthermore, serum sodium was 125 mEq/L (normal value: 135–145 mEq/L), calcium was 8.7 mg/dL (normal value: 8.5–10.5 mg/dL), blood albumin was 3.1 g/dL (normal value: 3.4–5.4 g/dL), and serum osmolarity was 292 mOsm/L (normal value: 275–295 mOsm/L).

**FIGURE 1:**
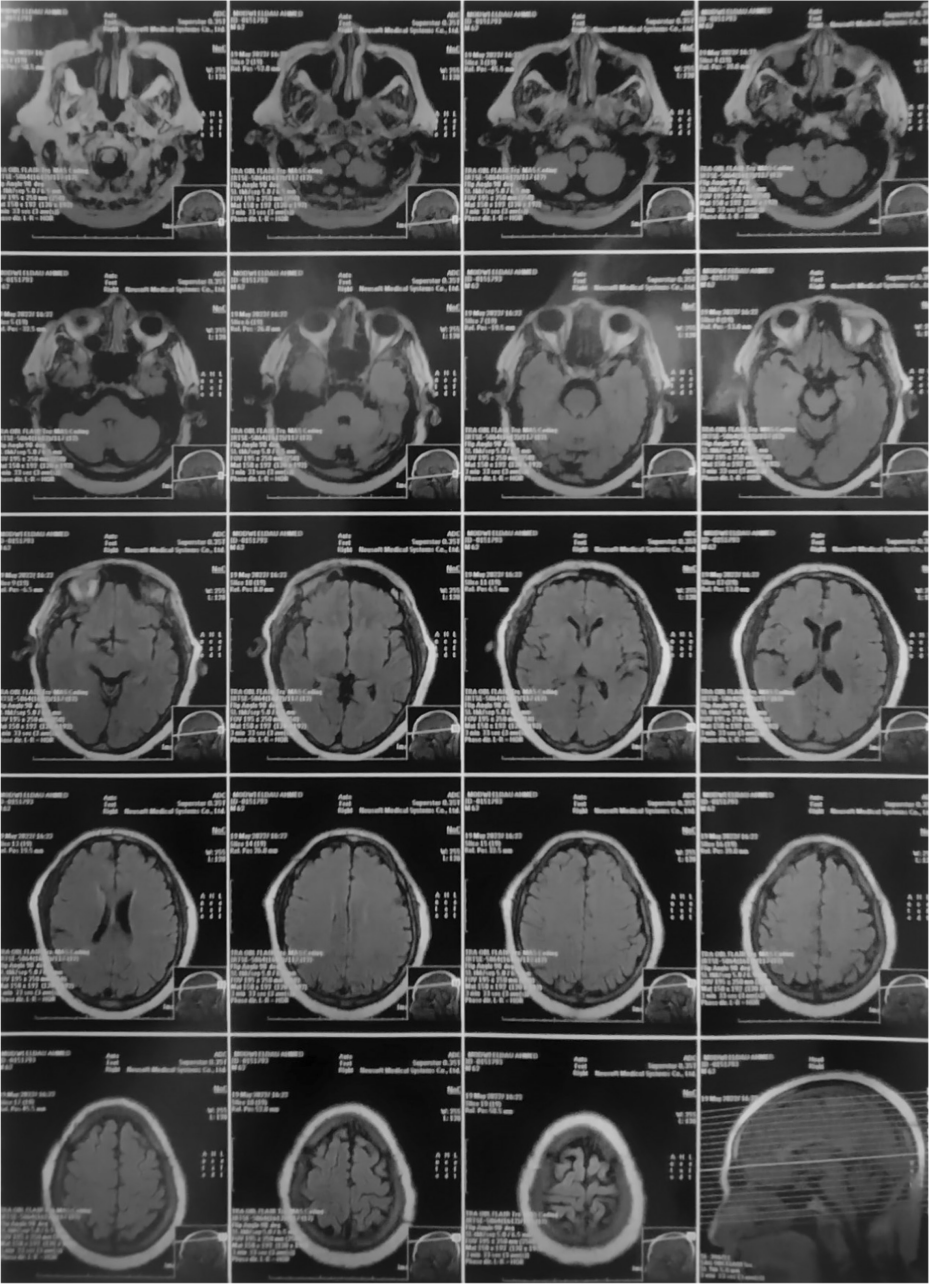
A Magnetic Resonance Image: The MRI shows the absence of lesions and structural abnormalities

The MRI (Figure 1), CSF, and EEG were of normal study. Based on that, the diagnosis of EPC was established.

The patients' diabetic ketoacidosis was treated by rehydration and correction of blood glucose. Additionally, sodium valproate was commenced, which adequately controlled the seizures. The patient was discharged 2 days later following the complete resolution of seizures and diabetic ketoacidosis. The list of discharge medications includes metformin and sodium valproate. The patient was referred to an endocrinologist for further follow‐up of DM. Three months later, upon subsequent follow‐up the patient did not suffer any following seizures.

## 3 DISCUSSION

EPC is characterized by repeated clonic muscular contractions confined to a single body part at a regular short intervals, persisting for days or weeks.^13^ Rarely hyperglycemia may lead to seizure, such as in cases of NKH. However, it is very rare in cases of DKA.^14^ There are only three case reports of EPC in patients with ketotic hyperglycemia.

In hyperglycemic crises, hyperglycemia may lower the gamma‐aminobutyric acid (GABA) levels, an inhibitory neurotransmitter, lowering the seizure threshold, and thus, explaining the occurrence of EPC.^15^ It is important to mention that ketosis and acidosis increase the seizure threshold; thus, seizures are not common in DKA.^16^

In our case, DKA was the first presentation of the DM. In addition, the MRI and CSF were normal. This is consistent with Van et al case report of EPC in a patient with DKA.^10^ In another case report of EPC with DKA, however, MRI showed hypointense lesions in the white matter below the left precentral gyrus which upon follow‐up imaging became hyperintense .^14^ Authors attributed this to gliosis.^14^ A previous study stated that mild focal gliosis may occur after a seizure.^17^

## 4 CONCLUSION

This report highlighted the association between EPC and diabetic ketoacidosis. Investigating metabolic disorders is an important part of assessing seizures, as EPC is resistant to anti‐epileptic as a sole treatment, detecting and treating the underlying cause is a cornerstone aspect of management.

## AUTHOR CONTRIBUTIONS

**Ahmed Sayedahmed:** Conceptualization; data curation; investigation; resources; software; writing – original draft; writing – review and editing. **Abdelrahman Mohammed Alkhair:** Data curation; investigation; writing – original draft; writing – review and editing. **Abdalla Mohamedali:** Software; writing – original draft; writing – review and editing. **Mahmoud Mahgoub:** Writing – original draft; writing – review and editing.

## CONFLICT OF INTEREST

The authors declare no conflict of interest. This case report did not receive any form of funding.

## ETHICAL APPROVAL

Patient confidentiality was maintained in this report, and all ethical considerations were done in accordance with the Declaration of Helsinki.

## CONSENT

A written informed consent was obtained from the patient prior to the writing and submission of this case report.

## Data Availability

The data that support the findings of this study are available from the corresponding author upon reasonable request.
